# Genomic Features for Desiccation Tolerance and Sugar Biosynthesis in the Extremophile *Gloeocapsopsis* sp. UTEX B3054

**DOI:** 10.3389/fmicb.2019.00950

**Published:** 2019-05-07

**Authors:** Catalina Urrejola, Jaime Alcorta, Loreto Salas, Mónica Vásquez, Martin F. Polz, Rafael Vicuña, Beatriz Díez

**Affiliations:** ^1^Department of Molecular Genetics and Microbiology, Pontificia Universidad Católica de Chile, Santiago, Chile; ^2^Laboratorio de Ecología Microbiana de Sistemas Extremos, Department of Molecular Genetics and Microbiology, Pontificia Universidad Católica de Chile, Santiago, Chile; ^3^Laboratorio de Ecología Microbiana y Toxicología Ambiental, Department of Molecular Genetics and Microbiology, Pontificia Universidad Católica de Chile, Santiago, Chile; ^4^Department of Civil and Environmental Engineering, Massachusetts Institute of Technology, Cambridge, MA, United States

**Keywords:** Atacama Desert, compatible solutes, cyanobacteria, desiccation tolerance, DNA extraction, exopolysaccharide, glycosyltransferase, trehalose

## Abstract

For tolerating extreme desiccation, cyanobacteria are known to produce both compatible solutes at intracellular level and a copious amount of exopolysaccharides as a protective coat. However, these molecules make cyanobacterial cells refractory to a broad spectrum of cell disruption methods, hindering genome sequencing, and molecular studies. In fact, few genomes are already available from cyanobacteria from extremely desiccated environments such as deserts. In this work, we report the 5.4 Mbp draft genome (with 100% of completeness in 105 contigs) of *Gloeocapsopsis* sp. UTEX B3054 (subsection I; Order Chroococcales), a cultivable sugar-rich and hardly breakable hypolithic cyanobacterium from the Atacama Desert. Our *in silico* analyses focused on genomic features related to sugar-biosynthesis and adaptation to dryness. Among other findings, screening of *Gloeocapsopsis* genome revealed a unique genetic potential related to the biosynthesis and regulation of compatible solutes and polysaccharides. For instance, our findings showed for the first time a novel genomic arrangement exclusive of Chroococcaceae cyanobacteria associated with the recycling of trehalose, a compatible solute involved in desiccation tolerance. Additionally, we performed a comparative genome survey and analyses to entirely predict the highly diverse pool of glycosyltransferases enzymes, key players in polysaccharide biosynthesis and the formation of a protective coat to dryness. We expect that this work will set the fundamental genomic framework for further research on microbial tolerance to desiccation and to a wide range of other extreme environmental conditions. The study of microorganisms like *Gloeocapsopsis* sp. UTEX B3054 will contribute to expand our limited understanding regarding water optimization and molecular mechanisms allowing extremophiles to thrive in xeric environments such as the Atacama Desert.

## Introduction

The Atacama Desert is the driest warm desert on Earth (Houston and Hartley, 2003; Hartley et al., 2005). Located in Northern Chile, for many years it was thought to be a sterile territory, unable to give shelter to any kind of living organism (McKay et al., 2003; Navarro-González et al., 2003). We have recently learned that in the Atacama Desert the occasional water inputs coming from the coastal fog and dew sustain the scarce microbial life thriving under its characteristic extreme environmental conditions (Houston and Hartley, 2003; McKay et al., 2003).

Microbial life has developed a variety of physical and molecular strategies to overcome the high solar radiation and temperatures, as well as to maximize the efficiency in the use of the low amount of water available. Indeed, most microbial life in deserts is somehow associated to rocks, developing either within or underneath them (Chan et al., 2012; Pointing and Belnap, 2012; Cowan et al., 2014; Davila et al., 2015; Wierzchos et al., 2015). These microbial communities are dominated by primary producers, represented mainly by morphological and metabolically diverse cyanobacteria (Pointing et al., 2009; Wong et al., 2010; Wierzchos et al., 2015; Wei et al., 2016). Most of these desert cyanobacteria produce copious extracellular structures, a feature that is thought to constitute both the architectural and metabolic basis for the microbial community and its tolerance to extreme environmental conditions (Knowles and Castenholz, 2008; Colica et al., 2014; Rossi and De Philippis, 2015).

Specifically, cyanobacteria organized in packet-like structures such as *Chroococcidiopsis* and *Gloeocapsa*, dominate lithic communities found in warm deserts (Warren-Rhodes et al., 2006; Bahl et al., 2011; Chan et al., 2012; Wierzchos et al., 2015; Crits-Christoph et al., 2016). Although these microorganisms have been identified by microscopy and by 16S-rDNA surveys, a significant barrier to progress in the study of cyanobacteria in general has been the difficulties encountered in obtaining axenic cultures, as well as the presence of copious exopolysaccharide (EPS) that hinders sequencing of their genomes (Tillett and Neilan, 2000; Chrismas et al., 2016). To illustrate the latter, only 1,110 from a total of 76,299 genomes available in the Integrated Microbial Genomes and Microbiomes (IMG/JGI) database correspond to cyanobacteria. Solely the marine genus *Prochlorococcus* concentrates a 55.85% of the cyanobacterial genomes already available. Moreover, merely three genomes correspond to cyanobacteria isolated from desert environments, all of which are filamentous.

An increasing body of knowledge supports the fundamental role that compatible solutes (Hershkovitz et al., 1991; Hill et al., 1994, 1997; Sakamoto et al., 2009; Yoshida and Sakamoto, 2009; Klähn and Hagemann, 2011) and EPS (Grilli Caiola et al., 1993, 1996; Hill et al., 1997; Tamaru et al., 2005; Knowles and Castenholz, 2008; Mager and Thomas, 2011) might play in desiccation tolerance in cyanobacteria. However, comprehensive genomic analyses of the mechanisms for tolerating extreme desiccation in unicellular cyanobacteria are still missing. In that sense, we decided to sequence and to study the genome of *Gloeocapsopsis* sp. UTEX B3054, a unicellular cyanobacterium that we demonstrate belongs to the Chroococcaceae family, and which possesses few known cultivable and sequenced representatives. This strain was obtained by cell-sorting from *Gloeocapsopsis* sp. AAB1 culture, an enrichment initially collected from a quartz rock in the Atacama Desert and described to be extremely tolerant to desiccation (Azúa-Bustos et al., 2014). Besides improving the genome coverage of this family of cyanobacteria, our study aimed to predict and analyze the genomic mechanisms likely associated to the desiccation tolerance of *Gloeocapsopsis* sp. UTEX B3054. In particular, we focused on identifying the genetic potential and genomic mechanisms likely involved in the biosynthesis of compatible solutes and EPS, molecules that play a key role in microbial tolerance to dryness.

## Materials and Methods

### Strain Isolation and DNA Extraction

The strain used in this study*, Gloeocapsopsis* sp. UTEX B3054, was obtained from the non-axenic *Gloeocapsopsis* sp. AAB1 enrichment culture initially isolated from the Atacama Desert (Azúa-Bustos et al., 2014). To massively eliminate contaminant heterotrophic bacteria, a single cyanobacterial cell was sorted into BG11 media using an Influx Mariner Cell Sorter (Cytopeia, Seattle, WA, United States). Chlorophyll-containing cells were detected based on red fluorescence (692–40 nm; fluorescence filters are specified here by the wavelength of maximum transmission and spectral width of bandpass) excited with a 488 nm laser, while triggering was based on side light scatter (SSC) to allow the exclusion of non-fluorescent cells. The clone of *Gloeocapsopsis* sp. UTEX B3054 culture was deposited in the UTEX Culture Collection of Algae under the accession code UTEX B3054, and it is publicly available.

Several treatments were further implemented in order to mechanically, chemically and enzymatically destabilize cyanobacterial aggregates, to selectively eliminate sugar molecules, and to eliminate persistent accompanying heterotrophic bacteria in culture. The first treatment was applied to healthy cultures in mid-active growth and was based on a previously reported protocol for sugar-rich cells (Sharma et al., 2002), with several modifications detailed in Supplementary Figure S1. In order to ensure degradation of remaining non-cyanobacterial DNA, the pellet of cyanobacterial cells obtained after the first treatment was resuspended in 100 μl of sterile water containing four units of DN*ase* I (Invitrogen) and incubated at 37°C for 1 h. The enzymatic reaction was stopped at 65°C using the DNase Stop solution (Invitrogen), and the pellet of cyanobacterial cells was three-time washed with sterile water. The last treatment aimed at breaking up cyanobacterial cells and to finally extract cyanobacterial DNA, and was based on the protocol described by Tillett and Neilan (2000) that was slightly modified adding a mechanical cell disruption step (glass beads and beadbeater) prior to DNA extraction.

### Genome Sequencing, Assembly, and Annotation

*Gloeocapsopsis* sp. UTEX B3054 DNA was used for genome sequencing with Illumina MiSeq. The data were analyzed using the Illumina pipeline 1.4.0 to generate fastq files. The raw sequences were cleaned of barcode, the quality was checked with FastQC software (Andrews, 2010) and reads were processed with Trimmomatic (Bolger et al., 2014). The trimmed reads were *de novo* assembled into 25,153 contigs with a total length of 18,225,500 bp using Spades v.3.9.0 (Bankevich et al., 2012). Contigs below 3,000 bp were discarded from the final pool of sequences since their closest nucleotide identity match was non-cyanobacterial followed by a second *de novo* assembly using only corresponding trimmed reads from contigs >3,000 bp. In this assembly, all contigs presenting low levels of depth sequencing matched to DNA sequences from heterotrophic bacteria and were therefore eliminated. CheckM tool v1.0.5 was used to calculate the completeness and quality (contamination grade) of the obtained genome (Parks et al., 2015). Closest available genomes were analyzed by Tetra Correlation Search (TCS) and by average nucleotide identity (ANI) using JSpecies tool (Richter et al., 2015).

Genome automatic annotation was carried out using the PROKKA v.1.11 software (Seemann, 2014), and it was submitted to JGI IMG/ER for annotation (GOLD Analysis Project ID: Ga0181813). Genome sequence and annotation data are available at the JGI IMG/ER database (GOLD Taxon ID: 2756170284; Sequencing Project: Gp0208497 and Analysis Project ID: Ga0181813). Further manual annotation was performed for a limited set of genes of interest, namely those involved in the biosynthesis of compatible solutes sucrose and trehalose, and envelope related genes. The search for these genes was carried out using: the automatic annotation results as query, but complementing them by performing BLASTP search (Altschul et al., 1997) for sequence homology using amino acid sequences of characterized bacterial orthologs of genes of interest (threshold used: *e*-value < *e*-10 and bitscore > 30). Besides, all putative ortholog genes found in *Gloeocapsopsis’s* genome were analyzed in the InterProScan database to corroborate the presence of specific functional domains. In the case of glycosyltransferase encoding genes, the classification into different sequence based-families was performed by following the classification available at the CAZy (Carbohydrate-Active enZymes) website (Coutinho et al., 2003). Finally, all the putative genes of interest found in *Gloeocapsopsis* sp. UTEX B3054 were used as query for a bidirectional BLASTP search against related cyanobacterial genomes (threshold used: *e*-value < 1*e*-15 and bit score > 100). In particular, in the case of genes putatively associated to trehalose biosynthesis and transport, automatic annotation identified several putative orthologs but with low levels of amino acid sequence identity (<35%). Thus, in order to confirm the protein homology, an additional three-dimensional protein structure prediction was performed in the Phyre2 webserver (Kelly et al., 2015), aiming to infer structural homology of the predicted proteins where certain functional domains were absent and/or when sequence identity was below 35%.

### Genome Analysis

The phylogenomic species tree was generated by a concatenation of amino acid sequences of 43 cyanobacterial marker genes (Parks et al., 2015). These sequences were extracted from *Gloeocapsopsis* sp. UTEX B3054 genome and other 74 cyanobacterial genomes available on the NCBI database by using the CheckM tool v1.0.5 (Parks et al., 2015). In the case of the *sug*ABC tree, sequences were also retrieved from the NCBI database and corresponded to 33 different genomes, including *Gloeocapsopsis* sp. UTEX B3054. Sequences were aligned using the MUSCLE v6.0.98 software (Edgar, 2004). Maximum-likelihood trees were generated with the IQtree v.1.5.5 software, with non-parametric ultrabootstrap support of 10,000 replicates (Hoang et al., 2017). For the multilocus tree as well as for TreH and SugABC trees, the best evolutionary substitution models were selected from sequence alignments with the Modelfinder option contained in IQTree Software.

Analyses of potential horizontal gene transfer were performed using the HGTector software (Zhu et al., 2014), considering 50% of amino acid identity and 80% of sequence coverage for BLASTP results. By using the AntiSMASH 4.2.0 web server (Blin et al., 2017), general secondary metabolite biosynthetic pathways were found. The search included ClusterFinder and whole genome PFAM analysis.

## Results

### Obtaining a Purified and Non-degraded Cyanobacterial DNA Suitable for Genome Sequencing

For retrieving a high-quality DNA (260/280 ratio > 1.8) amenable for genome sequencing of the sugar-rich *Gloeocapsopsis*, it was necessary to massively eliminate heterotrophic bacteria for obtaining a pure and clonal culture, which was performed by single cell sorting coupled to flow cytometry. However, even after subsequent streaking and plating on agar, the culture remained not axenic (not shown). Thereafter, a three-step procedure was developed, which aimed at (1) eliminating heterotrophic bacteria and destabilizing cyanobacterial aggregates; (2) eliminating of remaining heterotrophic bacterial sequences; and (3) finally breaking up of cyanobacterial cells to extract a pure cyanobacterial DNA (Supplementary Figure S2). The final step for DNA extraction allowed to effectively break cyanobacterial cells and to selectively eliminate remnant sugar molecules. This protocol led to a sample of non-degraded DNA with an evident dominance of cyanobacterial DNA suitable for sequencing (Supplementary Figures S2B,C).

### Genome Features and Phylogeny

After DNA sequencing, bioinformatics filtering steps discarded remaining non-cyanobacterial sequences (reflected in two discrete peaks of G+C content, at 43 and 68%), allowing us to differentially assemble the cyanobacterial genome with a completeness of 100 and 1.4% of sequence contamination, as detected by CheckM tool. The resulting genome size comprises 5,478,916 bp, in 105 contigs ranging from 3,290 to 356,189 bp. The N_50_ corresponds to 90,437 bp, with final genome coverage of 138.33X. The G+C content is 42.41%, represented in a single peak. Genome size and G+C content are similar to those of *Gloeocapsa* sp. PCC7438 (5.9 Mb and 42.45%, respectively), the closest sequenced cyanobacterium to *Gloeocapsopsis* sp. UTEX B3054. Automatic genome annotation with PROKKA software resulted in a total of 5,165 protein-coding sequences (CDS), 40 tRNAs, 3 rRNAs, and 3 CRISPR arrays. Among the former, 1,767 sequences correspond to hypothetical proteins. General features of the *Gloeocapsopsis’s* genome in comparison to other fully sequenced genomes of cyanobacteria are shown in Table 1.

**TABLE 1:** General features of *Gloeocapsopsis* sp. UTEX B3054 and other cyanobacterial genomes.

	*Gloeocapsopsis* sp.	*Synechocystis* sp.	*Anabaena* sp.	*Nostoc*	*C. thermalis*	*Gloeocapsa* sp.
	UTEX B3054	PCC6803	PCC7120	*punctiforme*	PCC7203	PCC7428
Genome size (bp)	5,478,916	3,947,019	7,211,789	9,059,191	6,689,401	5,882,710
DNA G+C content (%)	42.41	41.7	41.22	41.34	44.44	42.45
Total genes	5,165	3,708	6,130	6,690	5,975	5,254
tRNA	40	41	70	88	46	41
5S rRNA	1	2	4	4	3	2
16S rRNA	2	2	4	4	3	2
23S rRNA	2	2	4	4	3	2
CRISPR arrays	3	3	13	8	2	3
Total number of GTs	129	43	114	110	162	131
%GT/Total genes	2.5	1.2	1.9	1.6	2.7	2.5
Types of GT (diversity)	30	21	25	27	24	30

**FIGURE 1:**
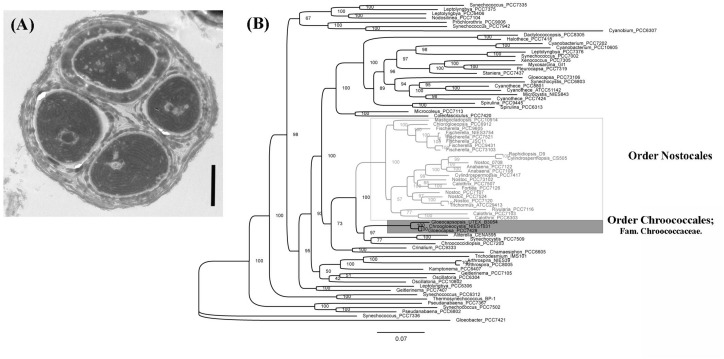
**(A)** Transmission electron photomicrograph of a characteristic tetrad of the unicellular *Gloeocapsopsis* sp. UTEX B3054. Note the multilayered and abundant extracellular polymer substance surrounding cells. Scale bar: 1 μm. **(B)** Phylogenomic tree generated by Maximum-likelihood using 43 cyanobacterial marker genes from 74 cyanobacterial genomes, based on the evolutionary substitution model LG+R8. *Gloeobacter violaceus* PCC7421 was used as outgroup. In gray box, the Chroococcaceae family (Order Chroococcales), which encompasses *Gloeocapsopsis* sp. UTEX B3054. This clade share ancestors with *Chroococcidiopsis thermalis* PCC7420, which has been assigned by Komárek et al. (2014) to the family Chroococcidiopsidaceae (order Chroococcidiopsidales). Few genomes of representatives of both clades have been successfully sequenced. Bootstrap values from phylogenetic analysis are displayed at the nodes. In white box, cyanobacterial representatives of section IV and section V. Both clades belong to the Nostocales order.

Phylogenomic analysis indicated that *Gloeocapsopsis* sp. UTEX B3054 formed a distinct clade with unicellular cyanobacteria *Gloeocapsa* sp. PCC7428 and *Chroogloeocystis siderophila* NIES-1031 (100% of node support and ∼82% of ANI) (Figure 1, in the gray box). The Chroococcaceae family (Order Chroococcales) represented here by *Gloeocapsopsis* sp. UTEX B3054 is well separated from, but basally related to, the also unicellular *Chroococcidiopsis thermalis* PCC7203 (97% of node support and 70.40% of ANI) (Figure 1). Our phylogenetic tree confirms the multicellular origin of these unicellular cyanobacteria.

Analysis with the HGTector software indicated that the number of potential CDS horizontally transferred was about 200, being the following the main bacterial order donors: Bacillales (15%), Ktedonobacterales (7%), Myxococcales (7%), and Rhizobiales (6%). Of these genes, 42% possess unknown functions, 35% are related to known metabolic pathways, and 16.5% are involved in information storage and processing. Genes related to the metabolism and transport of secondary metabolites, lipids, and saccharides are the most recurrent (Supplementary Table S1). Furthermore, 38 clusters encoding putative pathways of secondary metabolites were identified by the ClusterFinder method. Of them, 13 gene clusters might be associated with saccharide biosynthesis. Of particular interest are eight gene clusters found with the antiSMASH software, which encode for presumed biosynthetic pathways for bacteriocin (1), terpenes (1), aminoglycoside-aminocyclitol (1), polyketide synthase (PKS) (1), non-ribosomal peptide synthesis (NRPS) (1), and NRPS-PKS hybrids (3). Noteworthy, one of the latter is particularly extended (72.8 kbp), comprising almost 50 genes (Contig00007; location 61,046–133,879 nt). No complete homolog of this gene cluster was found in the IMG/JGI database, supporting its uniqueness. Overall, these eight genes clusters represent 4.92% (269,747 bp) of the whole genome of *Gloeocapsopsis* sp. UTEX B3054.

### Compatible Solutes: Sucrose and Trehalose Biosynthesis

Two genes involved in the biosynthesis of sucrose were found in *Gloeocapsopsis* sp. UTEX B3054 genome. The 2,420-bp gene of the sucrose-6-phosphate synthase enzyme (gene locus ID: BWI75_00738) was located in a genomic region devoid of other genes related to sugar metabolism. Its predicted amino acid sequence possesses the two characteristic enzymatic domains of sucrose synthase enzymes (InterProScan entries: IPR000368 and IPR001296), indicating that this protein belongs to the family 1 of glycosyltransferases (GTs). In contrast, the 806-bp gene of the sucrose-6-phosphate phosphatase enzyme (gene locus ID: BWI75_01845) was located upstream a gene cluster likely associated to sugar metabolism (gene locus ID’s: BWI75_01850 to BWI75_01856) containing three glycosyltransferase genes in tandem. Its predicted amino acid sequence contains a sucrose phosphatase domain (InterProScan entry: IPR006380) and a haloacid dehydrogenase HAD-like domain (InterProScan entry: IPR023214).

In turn, two putative genes encoding trehalose synthase protein TreS were identified (gene loci IDs: BWI75_02890 and BWI75_05160), sharing more than 50% of amino acid identity with a TreS of the desiccation-tolerant cyanobacterium *Leptolyngbya ohadii* (Murik et al., 2017). With 100% of confidence, three-dimensional predictions by the structure modeler Phyre2 indicated that BWI75_02890 and BWI75_05160 share 36 and 40% of sequence identity, respectively, with the trehalose synthase protein from *Mycobacterium smegmatis*, one of the most characterized trehalose synthase enzyme in the bacterial world (Ruhal et al., 2013). However, no homologous genes of *tre*Z (maltooligosyl-trehalose trehalohydrolase enzyme) neither *tre*Y (maltooligosyl-trehalose synthase) were identified in *Gloeocapsopsis* sp. UTEX B3054 genome.

**FIGURE 2:**
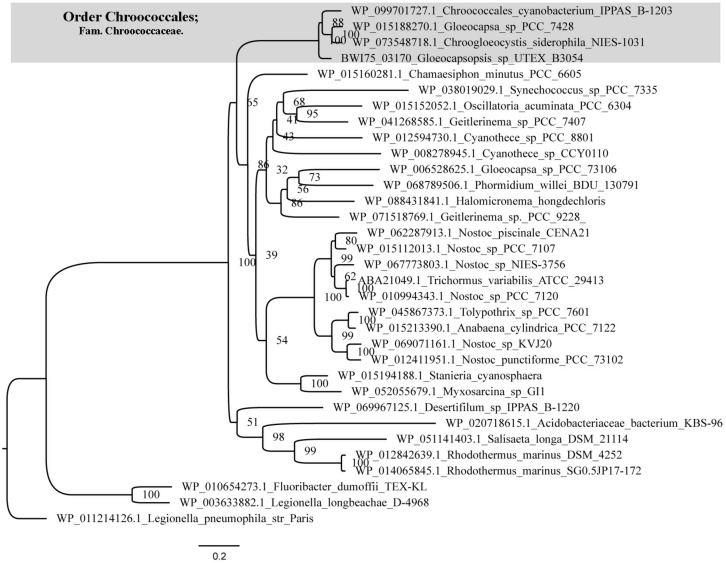
Phylogeny of cyanobacterial trehalase. Maximum-likelihood tree was generated with IQTree Software based on the evolutionary substitution model LG+I+G4. Trehalase proteins from the Chroococcaceae family (gray box) show an early divergence in comparison with Nostocales and other cyanobacteria. Number at the nodes indicate bootstrap values from phylogenetic analysis.

**FIGURE 3:**
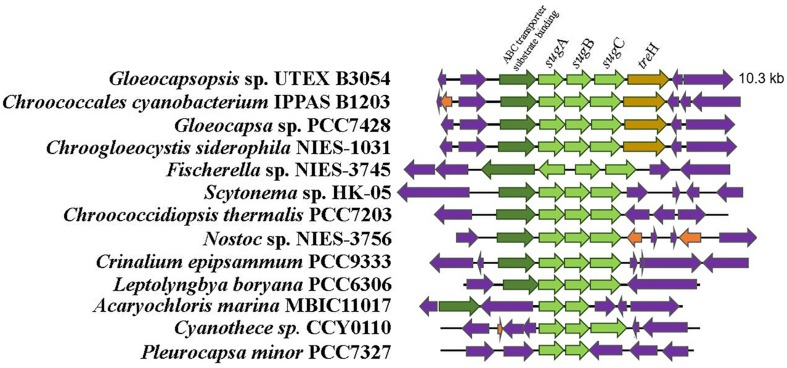
Organization of the *sug*A–*sug*B–*sug*C locus in cyanobacteria. Maximum-likelihood tree was generated with IQTree Software based on the evolutionary substitution model LG+I+G4. Only cyanobacteria from Chroococcaceae family have the *tre*H flanking the 3′ end of *sug*A–*sug*B–*sug*C genes. Note also that in this family an homoserine kinase and an ABC transport binding protein are located at the 5′ end of this gene cluster.

An ortholog of the *Anabaena* sp. PCC7120 trehalase (*tre*H) gene was also identified in *Gloeocapsopsis* genome (gene locus ID: BWI75_03170), sharing 63.87% of amino acid identity (alignment parameters: *e*-value 0.0; score 584). With 100% of confidence, three-dimensional predictions by the structure modeler Phyre2 indicated that BWI75_03170 shares 37% of sequence identity with the enzyme characterized as a neutral trehalase from the yeast *Saccharomyces cerevisiae*, Nth1. The phylogenetic reconstruction showed an early divergence of trehalase proteins from the Chroococcaceae family in comparison with related cyanobacteria like Nostocales (Figure 2). Interestingly, only in the Chroococcaceae family, *treH* gene is inserted in a conserved gene cluster comprising an ABC transporter system *sug*ABC (gene loci IDs: BWI75_03173; BWI75_03172; BWI75_03171, respectively) and a sugar binding protein located upstream of *treH* (gene locus ID: BWI75_ 03174), as well as a homoserine kinase (gene locus ID: BWI75_ 03175) (Figure 3).

### Envelope Related Genes: EPS Biosynthesis and Export

**FIGURE 4:**
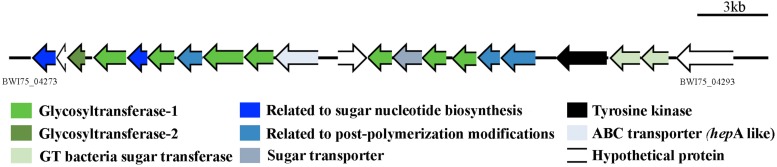
Gene cluster likely associated to desiccation tolerance mechanisms. This gene cluster contains 10 genes encoding for glycosyltransferases whose orthologs in *Anabaena* demonstrated to be overexpressed under desiccation conditions (Yoshimura et al., 2007). Gene locus: PWI75_04273 to PWI75_04293.

A genomic screening was conducted in order to identify all the putative glycosyltransferase (GT) proteins encoded by *Gloeocapsopsis* sp. UTEX B3054 and related cyanobacteria. A thorough search revealed that *Gloeocapsopsis* sp. UTEX B3054 possesses at least 129 genes encoding GTs (Table 1), a similar number to its closest evolutionary relative *Gloeocapsa* sp. PCC7428. *C. thermalis* PCC7203*, Gloeocapsa* sp. PCC7428 and *Gloeocapsopsis* sp. UTEX B3054 possess the highest GTs/CDS ratio (Table 1), in comparison to *Anabaena* sp. PCC7120, *Nostoc punctiforme*, and *Synechocystis* sp. PCC6803. *Gloeocapsopsis* sp. UTEX B3054 and *Gloeocapsa* sp. PCC7428 presented the highest levels of GT variety, with 30 different types. *Synechocystis* sp. PCC6803 contains the lowest diversity of GTs, encoding only 21 different types. The results indicate that most of the predicted cyanobacterial GTs are organized in functional modules, with conserved enzymatic motifs structured in tandem (Supplementary Tables S2–S6) and several different predicted domains combined in a large pool of putative GTs proteins. Most GTs found in *Gloeocapsopsis* sp. UTEX B3054 and other cyanobacterial genomes (above 70%) belongs to the GT1 and GT2 families (Supplementary Tables S2–S6). Many GT genes were found inserted within genomic clusters associated to sugar metabolism, such as in contig PWI75_000047 (Figure 4), which contains 10 GTs and additional genes associated to sugar metabolism, whose orthologs in *Anabaena* sp. PCC7120 are up-regulated under desiccation (Yoshimura et al., 2007).

The final step in extracellular polysaccharide biosynthesis comprises the assembly and translocation of the polysaccharide chains to the extracellular space. Three main pathways may carry out this step, which are the Wzy-dependent, the ABC-transporter dependent and the synthase dependent pathways (Pereira et al., 2009, 2015; Kehr and Dittmann, 2015). In *Gloeocapsopsis* sp. UTEX B3054, orthologs of genes associated with the three pathways for polysaccharide export were found (Table 2). In total, 223 genes representing 6.89% of the *Gloeocapsopsis* sp. UTEX B3054 genome are included in the cluster of orthologous gene (COG) category “Cell wall/membrane/envelope biogenesis”(not shown).

**TABLE 2:** Genes associated to EPS export pathways in *Gloeocapsopsis* sp. UTEX B3054.

EPS export pathway	Protein name	*Synechocystis* sp. PCC6803	Described function (Pereira et al., 2015)	*Gloeocapsopsis* sp. UTEX B3054	% ID	Alignment length	*E*-value	Score
Wzy-dependent pathway	Wzx	slr1543	Plasma membrane that translocate oligosaccharide lipid-linked	BWI75_01782	47.18	426	1,00E-97	301
		slr0896		BWI75_02201	53.48	445	4,00E-161	465
		slr0896		BWI75_00446	52.00	450	2,00E-149	436
		slr0896		BWI75_02497	51.66	453	2,00E-149	435
	Wzy	slr1515	Polymerize the oligosaccharide in the periplasm	BWI75_02924	50.68	444	3,00E-123	369
		slr0728		BWI75_00273	41.16	345	1,00E-67	220
	Wza	sll1581	Member of transenvelope complex	BWI75_01763	41.65	435	4,00E-108	332
	Wzb	slr0328	Phosphatase; control phosphorylation state of Wzc	BWI75_02935	50.00	132	6,00E-42	137
	Wzc	slr0923	Member of transenvelope complex	BWI75_04086	65.00	100	2,00E-46	145
ABC transporter- dependent pathway	KpsT	slr2108	Member of ATP transporter	BWI75_00465	53.89	347	3,00E-124	366
				BWI75_02053	43.04	230	2,00E-62	207
		slr0982		BWI75_02713	55.88	374	1,00E-125	372
		slr0982		BWI75_02053	51.98	202	3,00E-72	234
	KpsM	slr2107	Member of ATP transporter	BWI75_00464	67.18	259	4,00E-106	311
		slr0977		BWI75_02714	76.45	276	3,00E-129	370
Synthase-dependent pathway	Alg8	sll1377	Synthase involved in alginate production	BWI75_04753	56.20	468	5,00E-168	484
		sll1004		BWI75_04750	60.00	380	2,00E-152	438
	Alg44	sll1481	Binds c-di-GMP, regulating Alg8 function	BWI75_03665	40.61	421	7,00E-88	274
		sll1481		BWI75_00297	42.35	425	2,00E-82	260
		sll1481		BWI75_01048	40.86	421	7,00E-88	274
		sll1181		BWI75_04417	37.87	169	2,00E-25	107
		sll1181		BWI75_01316	40.49	536	2,00E-120	366
Undetermined	ExoD	slr1875	Participate in bacterial EPS production	BWI75_01173	54.07	209	4,00E-64	198

## Discussion

As in many cultivable cyanobacteria, *Gloeocapsopsis’s* axenity turned out as one of the major challenge. Initial attempts in our laboratory to sequence the *Gloeocapsopsis* genome confirmed the persistence of heterotrophic bacteria in the culture that prevented effective cyanobacterial genome sequencing. Thus, in a first effort for sequencing the AAB1 enrichment using 454 pyrosequencing technology, of a total of 19.8 Mbp obtained, a strikingly 95.5% corresponded to heterotrophic bacteria belonging to 12 different genera. In the present study, single cell sorting allowed us to overcome this situation, recovering a less contaminated clonal culture at least to a workable degree.

Our results demonstrated that all the technical efforts implemented in this study to effectively and to successfully deteriorate the extracellular polysaccharide structure to which heterotrophic bacteria are attached, reinforced the notion that *Gloeocapsopsis* sp. UTEX B3054 envelope is sugar-rich and impressively hard-to-break: cells were refractory to mechanical, physical, chemical, and biological disruption methods. Therefore, in molecular studies aiming at unveiling the microbial diversity and metabolic properties of extremophile microbes, the DNA extraction procedure should be of major concern (Lever et al., 2015).

The implementation of a three-step procedure allowed to differentially extract cyanobacterial DNA, obtaining a high-quality material for sequencing. This final protocol avoided the co-precipitation of sugars and contaminant DNA with the desired cyanobacterial DNA, which were likely interfering with DNA amplification by PCR, cloning efficiency, and further genome sequencing and assembly (Angeloni and Potts, 1987; Billi et al., 1998; Fiore et al., 2000; Tillett and Neilan, 2000; Chrismas et al., 2016). We estimate that this protocol might be adapted to other hard-to-lyse and sugar-rich non-axenic cyanobacteria.

Noteworthy, concomitant with the submission of this report, another group working with the original contaminated culture of *Gloeocapsopsis* released its genome (Puente-Sanchez et al., 2018). Although time-consuming, our cell isolation and DNA extraction protocols assisted us to obtain better genomic data, i.e., we obtained less contigs (105 vs. 137), and our shortest contig possesses 3,290 bp (vs. only 137 bp), whereas the longest contains 356,189 bp (vs. 250,842 bp). The N_50_ parameter of our assembly resulted to be higher (90,347 vs. 73,596). Moreover, due the fact that cyanobacteria possess genomes characterized by extended repetitive regions (Mazel et al., 1990; Asayama et al., 1996; Elhai et al., 2008), a customized DNA extraction protocol able to ensure high-quality DNA molecules is critical for facilitating the bioinformatics processing.

It is already known that cyanobacteria accumulate sucrose and trehalose not only in the cytoplasm, but in the extracellular matrix as a mechanism for tolerating desiccation (Sakamoto et al., 2009, 2011; Yoshida and Sakamoto, 2009; Azúa-Bustos et al., 2014; Murik et al., 2017). The genomic analysis of *Gloeocapsopsis* sp. UTEX B3054 revealed the presence of genes for sucrose and trehalose biosynthesis. Two forms of the enzyme sucrose phosphate synthase (SPS) have been found in cyanobacteria (Blank, 2013), and *Gloeocapsopsis* only encodes for an ortholog of the filamentous-related SPS, which possesses its characteristic single functional domain for glycosyl transferase.

Our genomic analysis revealed the presence of only one set of genes related to the biosynthesis of trehalose (the trehalose synthase genes) and the absence of *tre*Y/*tre*Z genes. *In silico* analysis predicted that trehalose synthase likely mediates the biosynthesis of trehalose in this cyanobacterium, whose treS homologs were transcriptionally up-regulated in the desiccation-tolerant *L. ohadii* subjected to simulated desiccation conditions (Murik et al., 2017). In both *Anabaena* sp. PCC7120 and *N. punctiforme*, trehalose concentration is modulated by TreH (Yoshida and Sakamoto, 2009), a trehalase enzyme which is encoded by a gene located within a gene cluster with maltooligosyl trehalose hydrolase (*tre*Y) and maltooligosyl trehalose synthase (*tre*Z) genes (Higo et al., 2006; Yoshida and Sakamoto, 2009). However, only in Chroococcaceae genomes, the *treH* gene was found near a putative sugar ABC-transporter gene cluster, suggesting the existence of a conserved mechanism for trehalose regulation specific for this family.

The ABC transporter associated with *tre*H gene of *Gloeocapsopsis* sp. UTEX B3054 possesses a conserved genomic arrangement and amino acid identity with the functionally described transporter LipqY-*Sug*ABC of *Mycobacterium tuberculosis* (27.00, 37.13, 38.70, and 42.93%, respectively). In *M. tuberculosis* this transporter possesses high specificity to trehalose and in conjunction with its periplasmic sugar-binding protein, works as an efficient retrograde recycling system for the disaccharide, participating actively in trehalose uptake and extracellular coat biosynthesis (Kalscheuer et al., 2010). Therefore, we hypothesize that cyanobacteria from Chroococcaceae family possess a novel retrograde recycling system for regulating trehalose concentration that might play a crucial role in preserving cells alive during extreme desiccation.

The most recent phylum-wide genomic study concerning EPS biosynthesis (Pereira et al., 2015) deliberately disregarded the study of GTs, in spite of the fact that they are thought to play a central role in bacterial polysaccharide biosynthesis (Schmid et al., 2016). These enzymes have been associated to envelope biosynthesis, including the EPS, the lipopolysaccharide and peptidoglycan, as well as to the glycosylation of membrane lipids and the biosynthesis of secondary metabolites, among other processes (Campbell et al., 1997; Coutinho et al., 2003).

The genome-wide analysis of GTs carried out in this work represents the first effort predicting the entire and diverse pool of GTs in cyanobacterial genomes. The whole classification of GTs is so far incomplete and might be improved as more GTs families become characterized. The information already available regarding envelope-related GTs in cyanobacteria is reduced and fractioned, and possibly overlooks an enormous diversity of putative GTs due to their large number and complexity in nature (Yang et al., 2007). The percentage of total encoded proteins that might be exclusively devoted to the synthesis of glycosidic bonds in *Gloeocapsopsis* resulted to be higher than the 1 to 2% of the total encoded proteins estimated for other genomes, whether archaeal, bacterial, or eukaryotes (Table 1; Lairson et al., 2008). Furthermore, *Gloeocapsopsis* sp. UTEX B3054 encodes ortholog genes of the three pathways already described in cyanobacteria for EPS transport and export (Pereira et al., 2015), suggesting that this cyanobacterium might possess the whole molecular machinery for translocation and export of polysaccharides to the extracellular space.

Several identified GTs were inserted within gene clusters comprising other genes related to sugar biosynthesis, as well as several gene clusters associated with saccharide biosynthesis related to secondary metabolites. For instance, the glycosyltransferase-rich gene cluster found in contig BWI75_000047 comes out as an attractive cluster for functional studies of desiccation tolerance mechanisms. This cluster contains genes whose corresponding orthologs are up-regulated in *Anabaena* sp. PCC7120 under desiccation (Yoshimura et al., 2007). Moreover, *Gloeocapsopsis* sp. UTEX B3054 possesses an ortholog of the RNA polymerase sigma factor SigJ (gene locus ID: BWI75_ 01223), described as a key regulator of desiccation tolerance in *Anabaena* sp. PCC7120 (68.58% of sequence identity) (Yoshimura et al., 2007). The longest gene cluster (Contig00007; location 61,046–133,879 nt) found by antiSMASH software came out also as a very interesting target for further functional studies: an PKS-saccharide-NRPS hybrid cluster comprising 72.8 kbp of unique genetic information that might potentially represent a new mechanism associated to the high tolerance this cyanobacterium possesses to extreme environmental conditions.

## Conclusion

The combination of single cell sorting and a customized multi-step DNA extraction protocol ensured the sequencing of the *Gloeocapsopsis* sp. UTEX B3054 genome. Technical difficulties encountered extracting nucleic acids confirmed the complexity of the extracellular matrix and the enormous content of sugar in this strain. Cells demonstrated to be hardly resistant to a wide spectrum of disruptive methods, highlighting the outstanding physicochemical properties of their protective coat.

The *in silico* analysis revealed the genetic potential to deal with water scarcity specific for *Gloeocapsopsis* and its relatives. Major efforts should be focused on deciphering the role of sugars during desiccation and specifically, the functional role that trehalase coupled to the ABC transporter might play controlling trehalose concentrations at both intra and extracellular levels. Moreover, the diversity of GTs found in this work suggests that the molecular complexity of the polysaccharide matrix might be potentially enormous.

We hope that the whole genomic framework provided in this work will help to untangle the sugar composition and structural arrangements of the cyanobacterial extracellular matrix, whose rheological properties seem to be critical for the retention of the meager water in Atacama.

## Author Contributions

CU, RV, and BD conceived the study. CU and LS conducted the experimental work and DNA extraction protocol. CU extracted the genomic DNA for sequencing under the guidance of MV, RV, and BD. BD planned the genome sequencing. MP conducted the genome sequencing. CU and JA conducted the genomic study under the supervision of BD. CU, JA, MV, RV, and BD analyzed the data. CU, RV, and BD wrote the manuscript. All authors read and commented on the drafted manuscript.

## Conflict of Interest Statement

The authors declare that the research was conducted in the absence of any commercial or financial relationships that could be construed as a potential conflict of interest.

## References

[B1] Altschul S. F. Madden T. L. Schäffer A. A. Zhang J. Zhang Z. Miller W. (1997). Gapped BLAST and PSI-BLAST: a new generation of protein database search programs. *Nucleic Acids Res.* 25 3389–3402. 9254694 10.1093/nar/25.17.3389 PMC146917

[B2] Andrews S. (2010). *FastQC a Quality Control Tool for High Throughput Sequence Data.* Available at: http://www.bioinformatics.babraham.ac.uk/projects/fastqc/ (accessed August 2016).

[B3] Angeloni S. V. Potts M. (1987). Purification of polysomes from a lysozyme- resistant desiccation-tolerant cyanobacterium. *J. Microbiol. Methods* 6 61–69. 10.1016/0167-7012(87)90054-90056

[B4] Asayama M. Kabasawa M. Takahashi I. Aida T. Shirai M. (1996). Highly repetitive sequences and characteristics of genomic DNA in unicellular cyanobacterial strains. *FEMS Microbiol. Lett.* 137 175–181. 10.1016/0378-1097(96)00050-X 8998982

[B5] Azúa-Bustos A. Zúñiga J. Arenas-Fajardo C. Orellana M. Salas L. Vicuña R. (2014). Gloeocapsopsis AAB1, an extremely desiccation-tolerant cyanobacterium isolated from the Atacama Desert. *Extremophiles* 18 61–74. 10.1007/s00792-013-0592-y 24141552

[B6] Bahl J. Lau M. C. Y. Smith G. J. D. Vijaykrishna D. Cary S. C. Lacap D. C. (2011). Ancient origins determine global biogeography of hot and cold desert cyanobacteria. *Nat. Commun.* 2 163–166. 10.1038/ncomms1167 21266963 PMC3105302

[B7] Bankevich A. Nurk S. Antipov D. Gurevich A. A. Dvorkin M. Kulikov A. S. (2012). SPAdes: a new genome assembly algorithm and its applications to single-cell sequencing. *J. Comput. Biol.* 19 455–477. 10.1089/cmb.2012.0021 22506599 PMC3342519

[B8] Billi D. Grilli Caiola M. Paolozzi L. Ghelardini P. (1998). A method for DNA extraction from the desert cyanobacterium Chroococcidiopsis and its application to identification of ftsZ. *Appl. Environ. Microbiol.* 64 4053–4056. 9758840 10.1128/aem.64.10.4053-4056.1998 PMC106599

[B9] Blank C. E. (2013). Phylogenetic distribution of compatible solute synthesis genes support a freshwater origin for cyanobacteria. *J. Phycol.* 49 880–895. 10.1111/jpy.12098 27007313

[B10] Blin K. Wolf T. Chevrette M. G. Lu X. Schwalen C. J. Kautsar S. A. (2017). AntiSMASH 4.0 - improvements in chemistry prediction and gene cluster boundary identification. *Nucleic Acids Res.* 45 W36–W41. 10.1093/nar/gkx319 28460038 PMC5570095

[B11] Bolger A. M. Lohse M. Usadel B. (2014). Trimmomatic: a flexible trimmer for Illumina sequence data. *Bioinformatics* 30 2114–2120. 10.1093/bioinformatics/btu170 24695404 PMC4103590

[B12] Campbell J. A. Davies G. J. Bulone V. Henrissat B. (1997). A classification of nucleotide-diphospho-sugar glycosyltransferases based on amino acid sequence similarities. *Biochem. J.* 326 929–939. 10.1042/bj2800309 9334165 PMC1218753

[B13] Chan Y. Lacap D. C. Lau M. C. Y. Ha K. Y. Warren-Rhodes K. A. Cockell C. S. (2012). Hypolithic microbial communities: between a rock and a hard place. *Environ. Microbiol.* 14 2272–2282. 10.1111/j.1462-2920.2012.02821.x 22779750

[B14] Chrismas N. A. M. Barker G. Anesio A. M. Sanchez-Baracaldo P. (2016). Genomic mechanisms for cold tolerance and production of exopolysaccharides in the Arctic cyanobacterium Phormidesmis priestleli BC1401. *BMC Genomics* 17:533. 10.1186/s12864-016-2846-4 27485510 PMC4971617

[B15] Colica G. Li H. Rossi F. Li D. Liu Y. De Philippis R. (2014). Microbial secreted exopolysaccharides affect the hydrological behavior of induced biological soil crusts in desert sandy soils. *Soil Biol. Biochem.* 68 62–70. 10.1016/j.soilbio.2013.09.017

[B16] Coutinho P. M. Deleury E. Davies G. J. Henrissat B. (2003). An evolving hierarchical family classification for glycosyltransferases. *J. Mol. Biol.* 328 307–317. 10.1016/S0022-2836(03)00307-303 12691742

[B17] Cowan D. A. Makhalanyane T. P. Dennis P. G. Hopkins D. W. (2014). Microbial ecology and biogeochemistry of continental Antarctic soils. *Front. Microbiol.* 5:154. 10.3389/fmicb.2014.00154 24782842 PMC3988359

[B18] Crits-Christoph A. Robinson C. K. Ma B. Ravel J. Wierzchos J. Ascaso C. (2016). Phylogenetic and functional substrate specificity for endolithic microbial communities in hyper-arid environments. *Front. Microbiol.* 7:301. 10.3389/fmicb.2016.00301 27014224 PMC4784552

[B19] Davila A. F. Hawes I. Araya J. G. Gelsinger D. R. DiRuggiero J. Ascaso C. (2015). In situ metabolism in halite endolithic microbial communities of the hyperarid Atacama Desert. *Front. Microbiol.* 6:1035. 10.3389/fmicb.2015.01035 26500612 PMC4594028

[B20] Edgar R. C. (2004). MUSCLE: multiple sequence alignment with high accuracy and high throughput. *Nucleic Acids Res.* 32 1792–1797. 10.1093/nar/gkh340 15034147 PMC390337

[B21] Elhai J. Kato M. Cousins S. Lindblad P. Costa J. L. (2008). Very small mobile repeated elements in cyanobacterial genomes. *Genome Res.* 18 1484–1499. 10.1101/gr.074336.107 18599681 PMC2527708

[B22] Fiore M. F. Moon D. H. Tsai S. M. Lee H. Trevors J. T. (2000). Miniprep DNA isolation from unicellular and filamentous cyanobacteria. *J. Microbiol. Methods* 39 159–169. 10.1016/S0167-7012(99)00110-114 10576706

[B23] Grilli Caiola M. Billi D. Friedmann E. I. (1996). Effect of desiccation on envelopes of the cyanobacterium Chroococcidiopsis sp. *Eur. J. Phycol.* 31 37–41.

[B24] Grilli Caiola M. Ocampo-Friedmann R. Friedmann E. I. (1993). Cytology of long-term desiccation in the desert cyanobacterium Chroococcidiopsis (Chroococcales). *Phycologia* 32 315–322. 11539431 10.2216/i0031-8884-32-5-315.1

[B25] Hartley A. J. Chong G. Houston J. Mather A. (2005). 150 million years of climatic stability: evidence from the Atacama Desert, Northern Chile. *J. Geol. Soc. Lond.* 162 421–424.

[B26] Hershkovitz N. Oren A. Cohen Y. (1991). Accumulation of trehalose and sucrose in cyanobacteria exposed to matric water stress. *Appl. Environ. Microbiol.* 57 645–648. 16348431 10.1128/aem.57.3.645-648.1991 PMC182773

[B27] Higo A. Katoh H. Ohmori K. Ikeuchi M. Ohmori M. (2006). The role of a gene cluster for trehalose metabolism in dehydration tolerance of the filamentous cyanobacterium Anabaena sp. *PCC* 7120. *Microbiology* 152 979–987. 10.1099/mic.0.28583-28580 16549662

[B28] Hill D. R. Keenan T. W. Helm R. F. Potts M. Crowe L. M. Crowe J. H. (1997). Extracellular polysaccharide of *Nostoc commune* (cyanobacteria) inhibits fusion of membrane vesicles during desiccation. *J. Appl. Phycol.* 9 237–248.

[B29] Hill D. R. Peat A. Potts M. (1994). Biochemistry and structure of the glycan secreted by desiccation-tolerant Nostoc commune (cyanobacteria). *Protoplasma* 182 126–148. 10.1007/BF01403474 10648523

[B30] Hoang D. T. Chernomor O. von Haeseler A. Minh B. Q. Le S. V. (2017). UFBoot2: improving the ultrafast bootstrap approximation. *Mol. Biol. Evol.* 35 518–522. 10.1093/molbev/msx281 29077904 PMC5850222

[B31] Houston J. Hartley A. (2003). The central Andean west-slope rainshadow and its potential contribution to the origin of hyper-aridity in the Atacama Desert. *Int. J. Climatol.* 23 1453–1464.

[B32] Kalscheuer R. Weinrick B. Veeraraghavan U. Besra G. S. Jacobs W. R. (2010). Trehalose-recycling ABC transporter LpqY-SugA-SugB-SugC is essential for virulence of Mycobacterium tuberculosis. *Proc. Natl. Acad. Sci. U.S.A.* 107 21761–21766. 10.1073/pnas.1014642108 21118978 PMC3003129

[B33] Kehr J. C. Dittmann E. (2015). Biosynthesis and function of extracellular glycans in cyanobacteria. *Life* 5 164–180. 10.3390/life5010164 25587674 PMC4390846

[B34] Kelly L. A. Mezulis S. Yates C. Wass M. Sternberg M. (2015). The Phyre2 web portal for protein modelling, prediction, and analysis. *Nat. Protoc.* 10 845–858. 10.1038/nprot.2015-2053 25950237 PMC5298202

[B35] Klähn S. Hagemann M. (2011). Compatible solute biosynthesis in cyanobacteria. *Environ. Microbiol.* 13 551–562. 10.1111/j.1462-2920.2010.02366.x 21054739

[B36] Knowles E. J. Castenholz R. W. (2008). Effect of exogenous extracellular polysaccharides on the desiccation and freezing tolerance of rock-inhabiting phototrophic microorganisms. *FEMS Microbiol. Ecol.* 66 261–270. 10.1111/j.1574-6941.2008.00568.x 18710394

[B37] Komárek J. Kaštovský J. Mareš J. Johansen J. R. (2014). Taxonomic classification of cyanoprokaryotes (cyanobacterial genera) 2014, using a polyphasic approach. *Preslia* 86 295–335.

[B38] Lairson L. L. Henrissat B. Davies G. J. Withers S. G. (2008). Glycosyltransferases: structures, functions, and mechanisms. *Annu. Rev. Biochem.* 77 521–555. 10.1146/annurev.biochem.76.061005.092322 18518825

[B39] Lever M. A. Torti A. Eickenbusch P. Michaud A. B. Šantl-Temkiv T. Jørgensen B. B. (2015). A modular method for the extraction of DNA and RNA, and the separation of DNA pools from diverse environmental sample types. *Front. Microbiol.* 6:476. 10.3389/fmicb.2015.00476 26042110 PMC4436928

[B40] Mager D. M. Thomas A. D. (2011). Extracellular polysaccharides from cyanobacterial soil crusts: a review of their role in dryland soil processes. *J. Arid Environ.* 75 91–97. 10.1016/j.jaridenv.2010.10.001

[B41] Mazel D. Houmard J. Castets A. M. Tandeau de Marsac N. (1990). Highly repetitive DNA sequences in cyanobacterial genomes. *J. Bacteriol.* 172 2755–2761. 2110150 10.1128/jb.172.5.2755-2761.1990 PMC208921

[B42] McKay C. P. Friedmann E. I. Gómez-Silva B. Cáceres-Villanueva L. Andersen D. T. (2003). Temperature and moisture conditions for life in the extreme arid region of the Atacama Desert: four years of observations including the El Niño of 1997 – 1998. *Astrobiology* 3 393–406. 14577886 10.1089/153110703769016460

[B43] Murik O. Oren N. Shotland Y. Raanan H. Treves H. Kedem I. (2017). What distinguishes cyanobacteria able to revive after desiccation from those that cannot: the genome aspect. *Environ. Microbiol.* 19 535–550. 10.1111/1462-2920.13486 27501380

[B44] Navarro-González R. Rainey F. A. Molina P. Bagaley D. Hollen B. J. de la Rosa J. (2003). Mars-like soils in the Atacama Desert, Chile, and the dry limit of microbial life. *Science* 302 1018–1021. 10.1126/science.1089143 14605363

[B45] Parks D. H. Imelfort M. Skennerton C. T. Hugenholtz P. Tyson G. W. (2015). CheckM: assessing the quality of microbial genomes recovered from isolates, single cells, and metagenomes. *Genome Res.* 25 1043–1055. 10.1101/gr.186072.114 25977477 PMC4484387

[B46] Pereira S. Zille A. Micheletti E. Moradas-Ferreira P. de Philippis R. Tamagnini P. (2009). Complexity of cyanobacterial exopolysaccharides: composition, structures, inducing factors and putative genes involved in their biosynthesis and assembly. *FEMS Microbiol. Rev.* 33 917–941. 10.1111/j.1574-6976.2009.00183.x 19453747

[B47] Pereira S. B. Mota R. Vieira C. P. Vieira J. Tamagnini P. (2015). Phylum-wide analysis of genes/proteins related to the last steps of assembly and export of extracellular polymeric substances (EPS) in cyanobacteria. *Sci. Rep.* 5:14835. 10.1038/srep14835 26437902 PMC4594306

[B48] Pointing S. B. Belnap J. (2012). Microbial colonization and controls in dryland systems. *Nat. Rev. Microbiol. Microbiol.* 10 551–562. 10.1038/nrmicro2831 22772903

[B49] Pointing S. B. Chan Y. Lacap D. C. Lau M. C. Y. Jurgens J. A. Farrell R. L. (2009). Highly specialized microbial diversity in hyper-arid polar desert. *Proc. Natl. Acad. Sci. U.S.A.* 106 19964–19969. 10.1073/pnas.0908274106 19850879 PMC2765924

[B50] Puente-Sanchez F. González-Silva C. Parro V. Tamames J. Azúa-Bustos A. (2018). Draft genome sequence of the extremely desiccation-tolerant cyanobacterium Gloecapsopsis sp. *strain AAB*1. *Genome Announc.* 5 1–2. 10.1128/genomeA.00216-18 29700137 PMC5920166

[B51] Richter M. Rosselló-Mora R. Glöckner F. O. Peplies J. (2015). JSpeciesWS: a web server for prokaryotic species circumscription based on pairwise genome comparison. *Bioinformatics* 32 929–931. 10.1093/bioinformatics/btv681 26576653 PMC5939971

[B52] Rossi F. De Philippis R. (2015). Role of cyanobacterial exopolysaccharides in phototrophic biofilms and in complex microbial mats. *Life* 5 1218–1238. 10.3390/life5021218 25837843 PMC4500136

[B53] Ruhal R. Kataria R. Choudhury B. (2013). Trends in bacterial trehalose metabolism and significant nodes of metabolic pathway in the direction of trehalose accumulation. *Microb. Biotechnol.* 6 493–502. 10.1111/1751-7915.12029 23302511 PMC3918152

[B54] Sakamoto T. Kumihashi K. Kunita S. Masaura T. Inoue-Sakamoto K. Yamaguchi M. (2011). The extracellular-matrix-retaining cyanobacterium *Nostoc verrucosum* accumulates trehalose, but is sensitive to desiccation. *FEMS Microbiol. Ecol.* 77 385–394. 10.1111/j.1574-6941.2011.01114.x 21507024

[B55] Sakamoto T. Yoshida T. Arima H. Hatanaka Y. Takani Y. Tamaru Y. (2009). Accumulation of trehalose in response to desiccation and salt stress in the terrestrial cyanobacterium *Nostoc commune*. *Phycol. Res.* 57 66–73. 10.1111/j.1440-1835.2008.00522.x 19436130

[B56] Schmid J. Heider D. Wendel N. J. Sperl N. Sieber V. (2016). Bacterial glycosyltransferases: challenges and opportunities of a highly diverse enzyme class toward tailoring natural products. *Front. Microbiol.* 7:182. 10.3389/fmicb.2016.00182 26925049 PMC4757703

[B57] Seemann T. (2014). Prokka: rapid prokaryotic genome annotation. *Bioinformatics* 30 2068–2069. 10.1093/bioinformatics/btu153 24642063

[B58] Sharma A. D. E. V. Gill P. K. Singh P. (2002). DNA isolation from dry and fresh samples of polysaccharide-rich plants. *Plant Mol. Biol. Rep.* 20 415a–415a.

[B59] Tamaru Y. Takani Y. Yoshida T. Sakamoto T. (2005). Crucial role of extracellular polysaccharides in desiccation and freezing tolerance in the terrestrial cyanobacterium *Nostoc commune*. *Appl. Environ. Microbiol.* 71 7327–7333. 10.1128/AEM.71.11.7327 16269775 PMC1287664

[B60] Tillett D. Neilan B. A. (2000). Xanthogenate nucleic acid isolation from cultured and environmental cyanobacteria. *J. Phycol.* 36 251–258.

[B61] Warren-Rhodes K. A. Rhodes K. L. Pointing S. B. Ewing S. A. Lacap D. C. Gómez-Silva B. (2006). Hypolithic cyanobacteria, dry limit of photosynthesis, and microbial ecology in the hyperarid Atacama Desert. *Microb. Ecol.* 52 389–398. 10.1007/s00248-006-9055-9057 16865610

[B62] Wei S. T. S. Lacap-Bugler D. C. Lau M. C. Y. Caruso T. Rao S. de los Rios A. (2016). Taxonomic and functional diversity of soil and hypolithic microbial communities in Miers Valley, McMurdo Dry Valleys, Antarctica. *Front. Microbiol.* 7:1642. 10.3389/fmicb.2016.01642 27812351 PMC5071352

[B63] Wierzchos J. DiRuggiero J. Vitek P. Artieda O. Souza-Egipsy V. Skaloud P. (2015). Adaptation strategies of endolithic chlorophototrophs to survive the hyperarid and extreme solar radiation environment of the Atacama Desert. *Front. Microbiol.* 6:934. 10.3389/fmicb.2015.00934 26441871 PMC4564735

[B64] Wong F. K. Y. Lacap D. C. Lau M. C. Y. Aitchison J. C. Cowan D. A. Pointing S. B. (2010). Hypolithic microbial community of quartz pavement in the high-Aatitude tundra of Central Tibet. *Microb. Ecol.* 60 730–739. 10.1007/s00248-010-9653-9652 20336290 PMC2974210

[B65] Yang Y. Qin S. Zhao F. Chi X. Zhang X. (2007). Comparison of envelope-related genes in unicellular and filamentous cyanobacteria. *Comp. Funct. Genomics* 2007:25751. 10.1155/2007/25751 18253473 PMC2211374

[B66] Yoshida T. Sakamoto T. (2009). Water-stress induced trehalose accumulation and control of trehalase in the cyanobacterium *Nostoc punctiforme* IAM M-15. *J. Gen. Appl. Microbiol.* 55 135–145. 10.2323/jgam.55.135 19436130

[B67] Yoshimura H. Y. Kamoto S. O. Sumuraya Y. T. Hmori M. O. (2007). Group 3 sigma factor gene, sigJ, a key regulator of desiccation tolerance, regulates the synthesis of extracellular polysaccharide in cyanobacterium Anabaena sp. *strain PCC* 7120. *DNA Res.* 14 13–24. 10.1093/dnares/dsm003 17376888 PMC2779892

[B68] Zhu Q. Kosoy M. Dittmar K. (2014). HGTector: an automated method facilitating genome-wide discovery of putative horizontal gene transfers. *BMC Genomics* 15:717. 10.1186/1471-2164-15-717 25159222 PMC4155097

