# Supplementary material for: Genomic Features for Desiccation Tolerance and Sugar Biosynthesis in the Extremophile Gloeocapsopsis sp. UTEX B3054
Source: Front Microbiol. 2019 May 7;10:950. doi: 10.3389/fmicb.2019.00950 (PMC6513891; doi:10.3389/fmicb.2019.00950)
Supplement: Supplementary file 1 [file Data_Sheet_1.pdf]

Figure S1. Implemented differential extraction protocol based on hexadecyltrimethyl ammonium bromide (CTAB) (modified from Sharma et al. (2002)).

- Resuspend cell pellet (washed cells obtained from the previous procedure) in 800 µl of NaCl 5M*. Mix by inversion, homogenizing the solution. Centrifuge at 3500 rpm. Carefully discard supernatant.

- Add 500 µl of bidistilled water, 30 µl of sodium dodecyl sulfate* (SDS) 10% and 3 µl of proteinase K* (20 mg/ml).

- Incubate samples at 55ºC, for one hour. (After this time, the samples should turn from a green to a brown color).

- Add 100 µl of NaCl 5M*. Mix by inversion.

- Add 200 µl of CTAB solution** (freshly made) previously heated at 65ºC. Incubate samples at 65ºC for 1 hour. **Modified CTAB solution**: Tris-HCl 100mM, pH 8.0; EDTA 20nM; NaCl 1.4M; CTAB (2% w/v); Polyvinylpyrrolidone (PVP) (3% w/v).

- Add glassbeads* (425-600 µm; Sigma-Aldrich) to the sample. Subject samples to mechanical disruption: three pulses of 30 seconds each in MiniLys beadbeater (Bertin Instruments).

- Add one volume of phenol:chloroform:isoamilic alcohol (25:24:1) to each tube. Mix by inversion.

- Centrifuge samples at maximum speed, for 20 minutes at room temperature (20-24ºC). This step should not be at these temperatures, avoiding sugar precipitation.

- Carefully transfer the supernatant to a new eppendorf tube; avoiding taking the interphase. Add 3 µl of RNase (10mg/ml) per 600 µl of supernatant. Incubate at 37ºC, 30 minutes.

- Add one volume of chloroform:isoamilic alcohol (24:1). Mix gently using vortex.

- Centrifuge samples at maximum speed and room temperature for 20 minutes. Transfer the supernatant to a clean eppendorf tube.

- Add 60 µl of sodium acetate 3M (1:10) and 450 µl of isopropanol. Mix slowly. Incubate at -20ºC at least one hour for DNA precipitation.

- Centrifuge samples at 4ºC and maximum speed. The DNA precipitate should be a white one in the bottom of the eppendorf tube. Discard supernatant.

- Wash the pellet with 80% ethanol. Dry the pellet and dissolve in pre-warmed sterile water.

* These steps aimed to disrupt cyanobacterial aggregates and to differentially extract DNA of persistent heterotrophic bacteria from the unialgal culture.
** CTAB is a cationic detergent used for facilitating the separation of polysaccharides, whereas the use of PVP aimed to absorb polyphenols that could potentially interfere in further molecular reactions.
