# Supplementary material for: Genomic Features for Desiccation Tolerance and Sugar Biosynthesis in the Extremophile Gloeocapsopsis sp. UTEX B3054
Source: Front Microbiol. 2019 May 7;10:950. doi: 10.3389/fmicb.2019.00950 (PMC6513891; doi:10.3389/fmicb.2019.00950)
Supplement: Supplementary file 2 [file Data_Sheet_2.PDF]

Figure S2 The multi-step DNA extraction protocol allowed to recover high-quality genomic DNA from *Gloeocapsopsis* sp. UTEX B3054.

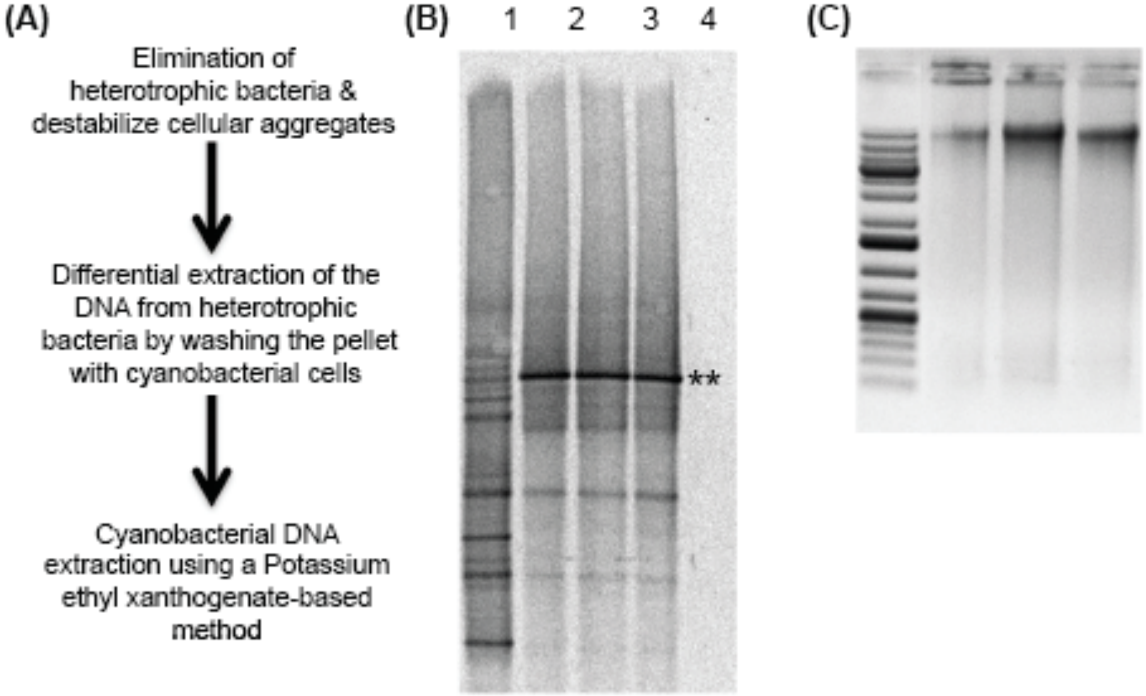

(A) Multiple-step procedure for effectively extracting cyanobacterial DNA from culture. **(B)** PCR-denaturing gradient gel electrophoresis (DGGE) analysis of the microbial diversity after a multiple-step DNA extraction procedure. Lane 1: DNA obtained from the first step by a CTAB-based method is used as indicator of microbial diversity or DNA contamination. Microbial diversity of the genomic sequences obtained after implementing the last DNA extraction step based on potassium-ethyl-xanthogenate are shown in lanes 2, 3 and 4 (replicates). Band highlighted with an asterisk correspond to the amplified 16S-rDNA from *Gloeocapsopsis* sp. UTEX B3054. The DDGE gel was constructed with a 30-50% denaturant gradient, and run in a Bio-Rad DCode^{TM} universal mutation detection system (Bio-Rad Laboratories, USA), at 60ºC and 100V for 16h, in 1X TAE buffer. After running, gels were stained with SybrGold® (Molecular Probes) for 30 min and then rinsed with MilliQ-purified water. **(C)** Agarose gel (1%) with genomic DNA obtained after the final step of the multiple-step DNA extraction protocol in replicates. Integrity of genomic DNA is evident and no smear is observed, indicator of DNA degradation and/or high content of sugar co-precipitated with nucleic acids. 1 Kb plus DNA ladder (Thermo Fisher Scientific) was used as standard.
