# Supplementary material for: Genomic Features for Desiccation Tolerance and Sugar Biosynthesis in the Extremophile Gloeocapsopsis sp. UTEX B3054
Source: Front Microbiol. 2019 May 7;10:950. doi: 10.3389/fmicb.2019.00950 (PMC6513891; doi:10.3389/fmicb.2019.00950)
Supplement: Supplementary file 3 [file Table_1.DOC]

**Table S1** List of genes horizontally transferred.

|  | ***Gloeocapsopsis sp. UTEX B3054*** |
| --- | --- |
| **INFORMATION STORAGE AND PROCESSING** | **33** |
| **[J]** Translation, ribosomal structure and biogenesis | 3 |
| **[A]** RNA processing and modification | - |
| **[K]** Transcription | 9 |
| **[L]** Replication, recombination and repair | 21 |
| **[B]** Chromatin structure and dynamics | - |
| **CELLULAR PROCESSES AND SIGNALING** | **13** |
| **[D]** Cell cycle control, cell division, chromosome partitioning | - |
| **[Y]** Nuclear structure | - |
| **[V]** Defense mechanisms | 2 |
| **[T]** Signal transduction mechanisms | - |
| **[M]** Cell wall/membrane/envelope biogenesis | 5 |
| **[N]** Cell motility | - |
| **[Z]** Cytoskeleton | - |
| **[W]** Extracellular structures | - |
| **[U]** Intracellular trafficking, secretion, and vesicular transport | - |
| **[O]** Posttranslational modification, protein turnover, chaperones | 6 |
| **METABOLISM** | **70** |
| **[C]** Energy production and conversion | 2 |
| **[G]** Carbohydrate transport and metabolism | 11 |
| **[E]** Amino acid transport and metabolism | 9 |
| **[F]** Nucleotide transport and metabolism | 3 |
| **[H]** Coenzyme transport and metabolism | 7 |
| **[I]** Lipid transport and metabolism | 12 |
| **[P]** Inorganic ion transport and metabolism | 7 |
| **[Q]** Secondary metabolites biosynthesis, transport and catabolism | 19 |
| **POORLY CHARACTERIZED** | **84** |
| **[R]** General function prediction only | - |
| **[S]** Function unknown | 84 |
| **TOTAL** | **200** |
| **PERCENTAGES** |  |
| INFORMATION STORAGE AND PROCESSING | **16,5** |
| CELLULAR PROCESSES AND SIGNALING | **6,5** |
| METABOLISM | **35,0** |
| POORLY CHARACTERIZED | **42,0** |
