# Supplementary material for: Genomic Features for Desiccation Tolerance and Sugar Biosynthesis in the Extremophile Gloeocapsopsis sp. UTEX B3054
Source: Front Microbiol. 2019 May 7;10:950. doi: 10.3389/fmicb.2019.00950 (PMC6513891; doi:10.3389/fmicb.2019.00950)
Supplement: Supplementary file 4 [file Table_2.DOC]

**TABLE S2.** Number of genes for glycosyltransferase enzymes belonging to family 1 in *Gloeocapsopsis* sp. UTEX B3054 and other closely related cyanobacteria.

|  | ***Synechocystis* sp. PCC6803** | ***Anabaena* sp. PCC7120** | ***Nostoc punctiforme*** | ***C. thermalis* PCC7203** | ***Gloeocapsa* sp. PCC7428** | ***Gloeocapsopsis* sp.** **UTEX B3054** | **Predicted protein domains (InterProScan)** | |
| --- | --- | --- | --- | --- | --- | --- | --- | --- |
| **GT family 1, subfamily 4** (Cell wall biosynthesis) | 0 | 2 | 3 | 1 | 1 | **1** | IPR028098 | |
|  | **12** | **30** | **36** | **56** | **38** | **29** |  | + IPR001296 |
|  | 0 | 0 | 0 | **1** | 0 | **0** |  | + IPR001296 + IPR001347 |
| **GT family 1**  (Mainly associated to sucrose, glycogen and starch biosynthesis) | **3** | **7** | **6** | **17** | **4** | **10** | IPR001296 | |
|  | 2 | 0 | 1 | 0 | 1 | **0** |  | + IPR022623 |
|  | 0 | 2 | 1 | 0 | 0 | **0** |  | + IPR013534 |
|  | 0 | 1 | 0 | 0 | 0 | **1** |  | + IPR000368 |
|  | **1** | 0 | 0 | 0 | 0 | **0** |  | + IPR000368 + IPR012822 + IPR012821 |

**IPR028098:** Glycosyltransferase, subfamily 4, N terminal domain.  **IPR001296:** Glycosyltransferase family 1 domain. **IPR001347:** Sugar isomerase domain. **IPR022623:** Domain of unknown function, DUF3495. **IPR013534:** Starch synthase, catalytic domain. **IPR000368:** Sucrose synthase domain.

**IPR012821:** Sucrose phosphate synthase, sucrose phosphatase-like domain. **IPR012822:** Sucrose phosphate synthase, glycosyltransferase domain.
