# Supplementary material for: Genomic Features for Desiccation Tolerance and Sugar Biosynthesis in the Extremophile Gloeocapsopsis sp. UTEX B3054
Source: Front Microbiol. 2019 May 7;10:950. doi: 10.3389/fmicb.2019.00950 (PMC6513891; doi:10.3389/fmicb.2019.00950)
Supplement: Supplementary file 5 [file Table_3.DOC]

**TABLE S3** Number of genes for glycosyltransferase enzymes belonging to family 2 in *Gloeocapsopsis* sp. UTEX B3054 and other closely related cyanobacteria.

|  | ***Synechocystis* sp. PCC6803** | | ***Anabaena* sp. PCC7120** | | ***Nostoc punctiforme*** | | ***C. thermalis* PCC7203** | | ***Gloeocapsa* sp. PCC7428** | | | ***Gloeocapsopsis* sp. UTEX B3054** | | **Predicted protein domains (InterProScan)** | |
| --- | --- | --- | --- | --- | --- | --- | --- | --- | --- | --- | --- | --- | --- | --- | --- |
| **GT family 2** (Combinations of several different functions with the sugar transference from UDP-glucose, UDP-N-acetyl-galactosamine, GDP-mannose or CDP-abequose, to a range of substrates including cellulose, dolichol phosphate and teichoic acids) | | **9** | | **37** | | **29** | | **42** | | **39** | **40** | | IPR001173 + IPR029044 | | |
|  |  | 0 | | 1 | | 0 | | 0 | | 0 | **1** | |  | | + IPR029063 |
|  |  | 0 | | 0 | | 0 | | 1 | | 0 | **1** | |  |  | + IPR027791 |
|  |  | 0 | | 0 | | 1 | | 2 | | 1 | **1** | |  |  | + IPR026461 |
|  |  | 1 | | 1 | | 1 | | 2 | | 1 | **1** | |  |  | + IPR011990 + IPR013026 |
|  |  | 0 | | 0 | | 0 | | 0 | | 0 | **1** | |  |  | + IPR008146 + IPR014746 |
|  |  | 1 | | 0 | | 1 | | 2 | | 0 | **0** | |  |  | + IPR007267 |
|  |  | 0 | | 0 | | **1** | | 0 | | 0 | **0** | |  |  | + IPR008638 + IPR011050 + IPR012334 + IPR024983 |
|  |  | 0 | | 1 | | 0 | | 0 | | 0 | **0** | |  |  | + IPR029489 |
|  |  | 0 | | 1 | | 0 | | 0 | | 0 | **0** | |  |  | + IPR017832 |
|  |  | 0 | | 0 | | 1 | | 0 | | 0 | **0** | |  |  | + IPR009875 |
|  |  | 0 | | 0 | | 0 | | 0 | | 1 | **0** | |  |  | + IPR009875  + IPR003919 |
|  |  | 0 | | 0 | | 0 | | 0 | | 1 | **0** | |  |  | + IPR017853 + IPR013781 + IPR006103 |
|  |  | 0 | | 0 | | 0 | | 1 | | 2 | **0** | |  |  | + IPR005150 + IPR003919 |

**IPR001173:** Glycosyltransferase family 2, domain. **IPR029044:** Nucleotide di-phospho-sugar transferase. **IPR029063:** S-adenosyl-L-methionine-dependent methyltransferase. **IPR027791:** Galactosyltransferase, C-terminal. **IPR026461:** Transferase 2, rSAM/selenodomain-associated. **IPR011990:** Tetratricopeptide-like, helical domain. **IPR013026:** Tetratricopeptide repeat-containing domain. **IPR008146:** Glutamine synthetase, catalytic domain. **IPR014746:** Glutamine synthetase/ guanidokinase, catalytic domain. **IPR007267:** GtrA-like protein. **IPR008638:** Filamentous haemagglutinin, N terminal. **IPR011050:** Pectin lyase factor. **IPR012334:** Pectin lyase fold.  **IPR024983:** CHAT (peptidase) domain.  **IPR029489:** O-GlcNAc transferase, C-terminal. **IPR017832:** Glycosyltransferase family 2, hopene-associated, hpnB. **IPR009875:** PilZ domain. **IPR017853:** Glycoside hydrolase superfamily. **IPR013781:** Glycoside hydrolase. **IPR006103:** Glycoside hydrolase family 2. **IPR005150:** Family of cellulose synthase. **IPR003919:** Cellulose synthase, subunit A.
