# Supplementary material for: Genomic Features for Desiccation Tolerance and Sugar Biosynthesis in the Extremophile Gloeocapsopsis sp. UTEX B3054
Source: Front Microbiol. 2019 May 7;10:950. doi: 10.3389/fmicb.2019.00950 (PMC6513891; doi:10.3389/fmicb.2019.00950)
Supplement: Supplementary file 6 [file Table_4.DOC]

**TABLE S4** Number of genes for glycosyltransferase enzymes belonging to families 2, 3 and 4 in *Gloeocapsopsis* sp. UTEX B3054 and other closely related cyanobacteria.

|  | ***Synechocystis* sp. PCC6803** | | ***Anabaena* sp. PCC7120** | | ***Nostoc punctiforme*** | | ***C. thermalis* PCC7203** | | ***Gloeocapsa* sp. PCC7428** | | | ***Gloeocapsopsis* sp. UTEX B3054** | | **Predicted protein domains (InterProScan)** | |
| --- | --- | --- | --- | --- | --- | --- | --- | --- | --- | --- | --- | --- | --- | --- | --- |
| **GT family 2**  (Mainly associated to capsular polysaccharide, cellulose and hopene biosynthesis) | | **1** | | **4** | | **7** | | **5** | | **2** | **2** | | IPR029044 | | |
|  |  | **1** | | 0 | | 0 | | 0 | | 0 | **0** | |  | | + IPR017832 |
|  |  | 0 | | 0 | | 0 | | 1 | | 1 | **1** | |  |  | + IPR002495 |
|  |  | 0 | | 0 | | 0 | | 0 | | 0 | **1** | |  |  | + IPR008441 |
|  |  | 0 | | 2 | | 0 | | 0 | | 0 | **2** | |  |  | + IPR019290 |
|  |  | 0 | | 1 | | 0 | | 0 | | 1 | **0** | |  |  | + IPR019290 + IPR025993 |
|  |  | **1** | | 0 | | 0 | | 0 | | 0 | **0** | |  |  | + IPR019290 + IPR025993 + IPR107835 |
|  |  | 0 | | 0 | | 0 | | 0 | | 0 | **1** | |  |  | + IPR019290 + IPR003919 + IPR009875 |
|  |  | 0 | | 0 | | 1 | | 1 | | 0 | **0** | |  |  | + IPR019290 + IPR005835 + IPR013446 |
| **GT family 3** (tTansfer phosphorylated ribose substrates) | | 0 | | 0 | | 0 | | 0 | | **1** | **0** | | IPR000312 + IPR017459 | | |
| **GT family 4**  (Phospho-N-acetylmuramoyl- pentapeptide-transferases) | | 0 | | 0 | | 1 | | 1 | | 0 | **0** | | IPR000715 | | |

**IPR029044:** Nucleotide di-phospho-sugar transferase. **IPR017832:** Glycosyltransferase family 2, hopene-associated, hpnB. **IPR002495:** Glycosyltransferase family 8. **IPR008441:** Capsular polysaccharide synthesis. **IPR019290:** Glycosyltransferase, family 2. **IPR025993:** Ceramide glycosyltransferase. **IPR107835:** Hopanoid biosynthesis associated glycosyltransferase, HpnI.  **IPR003919:** Cellulose synthase family. **IPR009875:** PilZ domain. **IPR005835:** Nucleotidyl transferase domain. **IPR013446:** Glucose phosphate cytidyltransferase.**IPR000312:** Glycosyltransferase family 3. **IPR017459:** Glycosyltransferase family 3, N-terminal. **IPR0000715:** Glycosyltransferase family 4.
