# Supplementary material for: Genomic Features for Desiccation Tolerance and Sugar Biosynthesis in the Extremophile Gloeocapsopsis sp. UTEX B3054
Source: Front Microbiol. 2019 May 7;10:950. doi: 10.3389/fmicb.2019.00950 (PMC6513891; doi:10.3389/fmicb.2019.00950)
Supplement: Supplementary file 7 [file Table_5.DOC]

**TABLE S5** Number of genes for glycosyltransferase enzymes belonging to families 9, 10, 14, 25 and 28 in *Gloeocapsopsis* sp. UTEX B3054 and other closely related cyanobacteria.

|  | ***Synechocystis* sp. PCC6803** | | ***Anabaena* sp. PCC7120** | | ***Nostoc punctiforme*** | | ***C. thermalis* PCC7203** | | ***Gloeocapsa* sp. PCC7428** | | | ***Gloeocapsopsis* sp. UTEX B3054** | | **Predicted protein domains (InterProScan)** | |
| --- | --- | --- | --- | --- | --- | --- | --- | --- | --- | --- | --- | --- | --- | --- | --- |
| **GT family 9**  (Transfer heptose to the lipopolysaccharide core) | | 1 | | 1 | | 1 | | 3 | | 4 | **2** | | IPR002201 | | |
| **GT family 10**  (Transfer fucose; fucosyltransferase) | | 0 | | 1 | | 1 | | 0 | | 0 | **0** | | IPR001503 | | |
| **GT family 14**  (Beta-1,6-N-acetylglucosaminyl transferase) | | 0 | | 0 | | 0 | | 0 | | 0 | **1** | | IPR003406 | | |
| **GT family 25**  (tTansfer of various sugars onto the growing lipopolysaccharide chain during its biosynthesis) | | **1** | | 0 | | 0 | | 0 | | 0 | **0** | | IPR002654 | | |
| **GT family 28**  (UDP N-acetylglucosaminyl- transferase) | | 1 | | 4 | | 0 | | 3 | | 4 | **3** | | IPR007235 | | |
|  |  | 0 | | 1 | | 0 | | 0 | | 1 | **1** | |  | | + IPR004276 |

**IPR002201:** Glycosyltransferase, family 9. **IPR001503:** Glycosyltransferase, family 10. **IPR003406:** Glycosyltransferase, family 14.

**IPR002654:** Glycosyltransferase, family 25. **IPR007235:** Glycosyltransferase, family 28, C-terminal.  **IPR004276:** Glycosyltransferase, family 28, N terminal.
