# Supplementary material for: Genomic Features for Desiccation Tolerance and Sugar Biosynthesis in the Extremophile Gloeocapsopsis sp. UTEX B3054
Source: Front Microbiol. 2019 May 7;10:950. doi: 10.3389/fmicb.2019.00950 (PMC6513891; doi:10.3389/fmicb.2019.00950)
Supplement: Supplementary file 8 [file Table_6.DOC]

**TABLE S6** Number of genes for glycosyltransferase enzymes belonging to different families in *Gloeocapsopsis* sp. UTEX B3054 and other closely related cyanobacteria.

|  | ***Synechocystis* sp. PCC6803** | | ***Anabaena* sp. PCC7120** | | ***Nostoc punctiforme*** | | ***C. thermalis* PCC7203** | | ***Gloeocapsa* sp. PCC7428** | | | ***Gloeocapsopsis* sp. UTEX B3054** | | **Predicted protein domains (InterProScan)** | |
| --- | --- | --- | --- | --- | --- | --- | --- | --- | --- | --- | --- | --- | --- | --- | --- |
| **GT family 39/83**  (Dolichyl-phosphate-mannose-protein mannosyltransferase and undecaprenyl phosphate-alpha-4-amino-4-deoxy-L-arabinose arabinosyl transferase proteins) | | 0 | | 0 | | 0 | | 1 | | 1 | **0** | | IPR003342 | | + IPR011990 |
|  |  | 0 | | 0 | | 0 | | 1 | | 1 | **1** | |  | | + IPR032421 |
| **GT family 51** (Use MurNAc-GlcNAc-P-P-lipid II as the sugar donor. And are responsible for the final stages of peptidoglycan biosynthesis for cell wall formation) | | 1 | | 4 | | 1 | | 0 | | 2 | **2** | | IPR001264 + IPR023346 | | + IPR001460 |
|  |  | 1 | | 1 | | 1 | | 1 | | 1 | **1** | |  | | + IPR000253 + **IPR012338** |
| **GT WecB/TagA/CpsF** (Involved in teichoic acid biosynthesis) | | 1 | | 2 | | 2 | | 7 | | 4 | **4** | | IPR004629 | | |
| **Bacterial sugar transferase**  (Capsular polysaccharide biosynthesis and diverse polysaccharide biosynthetis pathways) | | **1** | | **4** | | **5** | | **5** | | **3** | **8** | | IPR003362 | | |
|  |  | 1 | | 2 | | 1 | | 1 | | 4 | **2** | |  | | + IPR017475 |
|  |  | 1 | | 2 | | 1 | | 0 | | 1 | **0** | |  |  | + IPR002645 |
| **O-GlcNAc GT** (Transfer of a single GlcNAc to the Ser or Thr of proteins) | | **1** | | 0 | | 0 | | 0 | | 0 | **0** | | IPR029489 | | |
|  |  | 0 | | 1 | | 1 | | 0 | | 0 | **0** | |  | | + IPR011990 |
|  |  | 0 | | 0 | | 0 | | 0 | | 0 | **1** | |  |  | + IPR011990 + IPR013026 + IPR029063 |
| **UPD-GT**  (Transfer a glycosyl group from a UTP-sugar to a small hydrophobic molecule) | | 1 | | 0 | | 1 | | 0 | | 3 | **2** | | IPR002213 | | |
|  |  | 0 | | 0 | | 1 | | 0 | | 1 | **0** | |  | | + IPR004276 |
| **GT-Alg14**  (Dolichol-linked oligosaccharide biosynthesis) | | 0 | | 0 | | 0 | | 1 | | 2 | **2** | | IPR013969 | | |
| **GT AglD**  (Involved in S-layer biosynthesis) | | 0 | | 0 | | 0 | | 0 | | 1 | **1** | | IPR022791 | | |
| **GT** (Unclassified) | | 0 | | 1 | | 4 | | 6 | | 4 | **5** | | SSF53746; PF13692 | | |

**IPR003342:** Glycosyltransferase, family 39/83. **IPR011990:** Tetratricopeptide-like helical domain. **IPR032421:** O-mannosyl transferase, C-terminal. **IPR001264:** Glycosyltransferase, family 51. **IPR023346:** Lysozyme-like domain. **IPR001460:** Penicillin-binding protein, transpeptidase. **IPR000253:** Forkhead-associated domain. **IPR012338:** Beta-lactamase-transpeptidase like domain. **IPR004629:** Glycosyltransferase, Family WecB/TagA/CpsF. **IPR003362:** Bacterial sugar transferase. **IPR017475:** EPS biosynthesis polyprenyl glycosyl phospho transferase. **IPR002645:** Sulphate transporter and anti-sigma factor antagonist (STAS) domain. **IPR029489:** O-GlnNAc transferase, C-terminal. **IPR011990:** Tetratricopeptide-like helical domain. **IPR013026:** Tetratricpeptide repeat-containing domain. **IPR029063:** S-adenosyl-L-methionine-dependent methyltransferase. **IPR002213:** UDP-glucoronosyl/ UPD-glucosyltransferase.  **IPR004276:** Glycosyltransferase, family 28, N terminal. **IPR013969:** Oligosaccharide biosynthesis protein, Alg14-like. **IPR022791:** Lysophosphatidylglycerol synthetase/glycosyltransferase AglD domain. **SSF53746 and PF13692:** Unintegrated signatures in InterProScan database. Members of these families transfer UDP, ADP, GDP or CMP linked sugars to a variety of substrates.
